# Biosynthesis of zinc oxide nanoparticles using *Phoenix dactylifera* and their effect on biomass and phytochemical compounds in *Juniperus procera*

**DOI:** 10.1038/s41598-021-98607-3

**Published:** 2021-09-27

**Authors:** Abdalrhaman M. Salih, Fahad Al-Qurainy, Salim Khan, Mohamed Tarroum, Mohammad Nadeem, Hassan O. Shaikhaldein, Abdel-Rhman Zakaria Gaafar, Norah S. Alfarraj

**Affiliations:** Botany and Microbiology Department, College of Science King Saud University, P. O. BOX 2455, Riyadh, 11451 Saudi Arabia

**Keywords:** Biological techniques, Biotechnology

## Abstract

Biosynthesized nanoparticles have played vital role recently, as suggested to be alternative to physical and chemical methods. In this study, biosynthesis of zinc oxide nanoparticles (ZnO NPs) were carried out using leaf extracts of *Phoenix dactylifera L.* and Zinc nitrate. The effect of ZnO nanoparticles on biomass and biochemical parameters was investigated. Biosynthesized ZnO nanostructure was characterized using X-ray diffraction (XRD), transmission electron microscopy (TEM), UV–visible spectrophotometer and Fourier transform infrared spectroscopy (FTIR). Which resulted in spherical shape with size ranging between 16 to 35 nm of Biosynthesized ZnO nanoparticles and UV absorption beak at 370.5 nm with clear peaks of functional groups. The impact of different concentrations (0.0 mg/L, 80 mg/L and 160 mg/L) of biosynthesized ZnO nanoparticles on biomass and bioactive compounds production of *Juniperus procera *in vitro was investigated. The results showed that, biosynthesized ZnO NPs (80 mg/L and 160 mg/L) concentrations were boosted the growth of *J. Procera* with significantly compared to non-treated plants in vitro. The highest concentration (160 mg/L) of ZnO NPs was enhanced the growth of plant at beginning period, one month later shoots became yellow and callus turned to be brownish. Moreover, the influence of ZnO NPs on phytochemical compounds in callus of *Juniperus procera* was examined using GC–MS analysis. The differences among treatments were recoded**.** Overall, zinc oxide nanoparticles substantially improved the growth of shoots and callus with increasing of biochemical parameters such as chlorophyll a, total phenolic and flavonoids contents, besides the total protein and, SOD, CAT and APX activity. ZnO NPs might be induced some phytochemical compounds as well as inhibit.

## Introduction

Nanotechnology has become a new field of research, that dealing with synthesis of nanomaterials and nanoparticles for their applications in various fields such as catalysis, electrochemistry, biomedicines, pharmaceutics, and food technology, etc.^1–4^. The particles within the size less than 100 nm are known as nanoparticles (NPs) and it would be divided into different classes depend on their shapes and size as fullerenes, ceramic NPs, metal NPs and polymeric NPs^5,6^. There are many methods have been used for fabrication of nanoparticles, which include chemical and physical methods such as photochemical, radiation, chemical precipitation methods. These methods are non-environmental friendly due to the use of toxic, combustible, and hazardous chemicals and extremely expensive^7,8^. In contrast, plant extracts, fungi, and microbes mediated the synthesis process of NPs, it consider to be a suitable alternative method to non-environmental methods^9–11^. This method has been called ‘green synthesis or biosynthesis’ which is a less hazardous process than physical and chemical synthesis methods and low-cost^12^. It has been reported that, biosynthesized Zinc oxide nanoparticles are eco-friendly, which is provide many advantages such as antimicrobial activity against microorganisms, drug delivery and anticancer therapy as well^13^. A few studies have been conducted and tested the role of biosynthesized zinc oxide nanoparticle on plants biomass and bioactive compounds production in vitro. For example, ZnO NPs with low concentrations, it stimulated the callus growth and pointed out the nanoparticles role in regeneration, decontamination, organogenesis, callus induction and activated a protein that has a vital role in growth recounted by^14,15^. Previous studies have been provided evidence for NP-mediated modulation of plant secondary metabolism, beside that the studies provide an indirect link between secondary metabolism and reactive oxygen species^16^. The exposure of plants to NPs, has the potential to induce secondary metabolites of plants, and it act as phytoalexins to protect plants from biotic and abiotcs stress^16,17^. *Juniperus procera* is medicinal plant, widely spread throughout southern part of Saudi Arabia, and it is indigenous to the mountains of eastern Africa from east Sudan to Zimbabwe, and southwest of the Arabian Peninsula^18^. *Juniperus procera* is a source of natural drugs with potential for antimicrobial, anticancer, insecticidal antioxidant activities^19–21^. In nature, plants produce secondary metabolites as a protection mechanism. On the other hand, secondary metabolites can be produced and improved using micro propagation technique which is a reliable approach. Moreover, mass propagation is a rapid approach for production of important secondary metabolites^22–24^. Furthermore, plants have an important natural products have been used by human as condiments and flavorings and for treating health disorders and preventing diseases, including epidemics^25^. So far, natural products have made the basis for many useful agrochemicals, pharmaceuticals, and can be a alternative source for bioactive compounds to control several diseases in both crops and humans^26,27^. To our knowledge, there are no reports to date involving zinc oxide nanoparticles effect on biomass and bioactive compounds of *J. Procera *in vitro*.* Therefore, the main objective of the present study was to synthesis zinc oxide nanoparticles biologically, and to investigate their effect on biomass and bioactive compounds production from *Juniperus procera *in vitro. In this present study different characterization techniques such as TEM, XRD, FTIR and UV–Visible were used to investigate the formation of ZnO nanoparticles. Whereas, the effect of biosynthesized ZnO NPs on biomass and bioactive compounds of *J. Procera *in vitro was tested by the estimation of the total protein content and enzymes activity, and total phenolic content, flavonoids, and bioactive compounds.

## Material and methods

### Biosynthesis of ZnO NPs

Leaves *of Phoenix dactylifera* were collected from botanic garden, Dept. of Botany and Microbiology, King Saud University, with full permission has obtained from institute. The extract was prepared by drying leaves at room temperature and washed in distilled water, 5 g of this powder was homogenized completely in 100 ml Milli-Q water and extracted at ≤ 80 °C for 20 min. The resultant was filtered using Whatman filter papers No. 1. Then, extract was stored at 4 °C and used for generating biosynthesized zinc oxide nanoparticles. Zinc nitrate (99.999%) was purchased from Sigma. The synthesis of ZnO NPs was carried out by taking a 0.05 M of zinc nitrate in 100 ml Milli-Q water. Then, 2:2 (v/v) of leaf extract and zinc nitrate to obtain a mixture solution in a round-bottom flask, and incubated with constant stirring (100 rpm) at 40 °C for 24 h. The solution was cooled to room temperature and filtered using Whatman filter papers No. 1. The precipitate was washed with deionized water and absolute ethanol for several times using centrifugation (5000 rpm for 5 min), and dried in an oven at 60 °C for 24 h. Finally, the product was calcined at 600 °C for 3 h^28^.

### Characterization of ZnO NPs

The surface morphology and particle size of the ZnO nanostructure were investigated using transmission electron microscope (TEM). The crystalline structure of ZnO NPs was determined using X-ray diffractometer with Cu Kα radiations (λ = 1.5406 Å) operated at voltage of 40 kV and current of 15 mA. Fourier transmission infrared (FTIR) spectra of the powder was recorded using a Fourier transmission infrared spectrometer (Perkin Elmer) in the range of 5000–100 cm^−1^. Room temperature optical absorption spectrum was recorded in the range of 200–800 nm using a UV–Vis spectrophotometer (UV-1800, SHIMADZU, Japan).

### Plant material

*Juniperus procera* was in vitro regenerated by protocol which has made and developed in our laboratory, previously^29^ with full permission has obtained from institute. Firstly, cutting about 1 cm contained at least one axillary of *J. procera* were used as explants for in vitro propagation.

### Media preparation and nanoparticles treatment

Woody Plant Media (WPM) with supplement of Plant Growth Regulators (PGRs) 2,4-D and BAP (0.5 µM), sucrose as carbon source (30 g/L), 7 g/L of agar, and the pH was adjusted to 5.7. Then, Biosynthesized ZnO NPs different concentrations (0.0 mg/L, 80 mg/L and 160 mg/L) were added to the media before autoclaving at 121 °C for 20 min. Then, for each treatment triplicate with four explants per jar were cultured under laminar conditions. The jars were incubated in growth chamber at 25 °C ± 1, with 14/10 h illumination periods for 70 days.

### Chlorophyll determination

Chlorophyll content in the leaves of *J. procera* was carried out with a mixture of acetone and water at a ratio of 80% − 20% (v/v). 0.1 g of fresh leaves homogenized in 2 ml acetone solution 80%. Then, were stored at − 4 °C for 24 h. The mixture was centrifuged at 13000 rpm for 10 min. Absorption was measured at 663 and 645 nm using a UV-1800 spectrophotometer (Shimadzu, Japan). Estimation of chlorophyll a, b was carried out using Arnon method^30^.

### Estimation of the total protein content and enzymes activity

300 mg of callus were homogenized in liquid nitrogen and dissolved in 100 mM sodium phosphate buffer (pH 7.4) containing 1% PVP, and 0.5% (v/v) Triton-X 100. Then, homogenate was centrifuged at 20,000rmp for 20 min at 4 °C. Supernatant was collected and storetd at − 20 for determination of protein by Nano drop and specific activities of antioxidant enzymes were extracted and estimated as the methods described by^31^.

Superoxide dismutase (SOD, EC 1.15.1.1) activity was estimated using the method of Marklund and Marklund^32^. The reaction mixture has contained 1 mL of 0.25 mM pyrogallol, 1.9 mL of 0.1 M sodium phosphate buffer (pH 7.4), and 100 μL of enzyme extract. The absorbance was measured at 420 nm. The SOD activity (U g − 1 protein) was defined as the amount of enzyme needed for 50% inhibition of pyrogallol oxidation.

The catalase (CAT, EC 1.11.1.6) activity was estimated by measuring the absorbance at 240 nm, as per the method described by^33^. 1 mL of 0.059 M H2O2 in 0.1 M sodium phosphate buffer (pH 7.4), 1.9 mL of distilled water, and 100 μL of enzyme extract. The CAT activity was expressed as unit g − 1 of protein.

Ascorbate peroxidase (EC 1.11.1.11) activity was determined as the methods described by^34^. The reaction medium contained 1 mL of 0.1 M sodium phosphate buffer (pH 7.4), 1 ml distilled water, 100 µL ETDA (0.1 mM), hydrogen peroxide (100 µL), and an enzyme extract (100 µL). The absorbance was recorded at 290 nm, with 3 replicates.

### Proline measurement

Proline was extracted following the method described by ^35^. Liquid nitrogen has been used to grind fresh sample (0.5 g) and the product was extracted in 10 ml of 3% aqueous sulfo-salicylic acid. The mix was centrifuged and 2 ml of supernatant was added to 2 mL ninhydrin plus 2 mL glacial acetic acid. The mixture was boiled at 100 °C for 1 h, then the reaction was stopped by transferring the tubes to an ice bath for 5 min. Subsequently, 6 ml of toluene was added, mixed vigorously for 15 s and the absorbance of the upper phase was read at 520 nm. The proline content was expressed in μg/g fresh weight.

### Estimation of the total flavonoids

Estimation of the total flavonoids in the callus of *J. procera* extracts was carried out using the method describe by^36^. 0.5 mL of methanol extract, a volume of 0.5 mL of 2% AlCl3 water solution was added. After 24 h at room temperature, the absorbance was measured at 420 nm. A calibration curve was constructed, using quercetin (50–0400 µg/ml) as standard. Total flavonoid contents were expressed as quercetin (mg/g.dry wt.) using the following equation based on the calibration curve (y = 0.0014x + 0.0595).

### Estimation of total phenolic content

The total phenolic content of the callus of *J. procera* extract was determined by using Folin-Ciocalteu reagent following method described by Ainsworth^37^. Gallic acid was used as a reference standard calibration curve. A volume of 0.5 mL of the plant extract (100 µg/mL) was mixed with 2 mL of the Folin-Ciocalteu reagent (diluted 1:10 with de-ionized water) and were neutralized with 4 mL of sodium carbonate solution (7.5%, w/v). The reaction mixture was incubated at room temperature for 30 min. The absorbance of the resulting blue color was measured at 765 nm using UV–VIS spectrophotometer (SHIMADZU, UV − 1800). The total phenolic contents were determined from the linear equation of a standard curve prepared with Gallic acid. The content of total phenolic compounds expressed as mg/g gallic acid equivalent (GAE) of dry extract.

### Preparation of callus extracts for GC–MS analysis

50 mg of callus of *J. procera* were lyophilized before grinding. Then, has been extracted in 2.0 ml of methanol of 99.98% using Tissue Layser LT (Qiagen.) Voltage 24VDC/ power 40 VA for 2 h at 25 °C. The organic and aqueous phases were separated by centrifugation at 5000 rpm for 15 min. Then, supernatant was filtered using 0.45 µm nylon syringe before injected into GC–MS analysis.

### Statistical analysis

All experiments were done in triplicate and the results were reported in the figures and tables are the average of three replicate ± standard deviations. The statistical software SPSS (version 20) one-way ANOVA was used for evaluating statistical significance and at (*P* < 0.05).

### Legal statement

The collection of plant materials which are used in this study complies with relevant institutional, national, and international guidelines and legislation. Seeds and seedlings of the date palm and African pencil cedar were collected and provided by Botany and Microbiology Department (Garden and Herbarium Unit), College of Science, King Saud University (KSU) with full permission to collect plant materials by accepting the terms and conditions of national and international standards.

## Results and discussion

### Biosynthesis and characterization of ZnO nanoparticles

**Figure 1:**
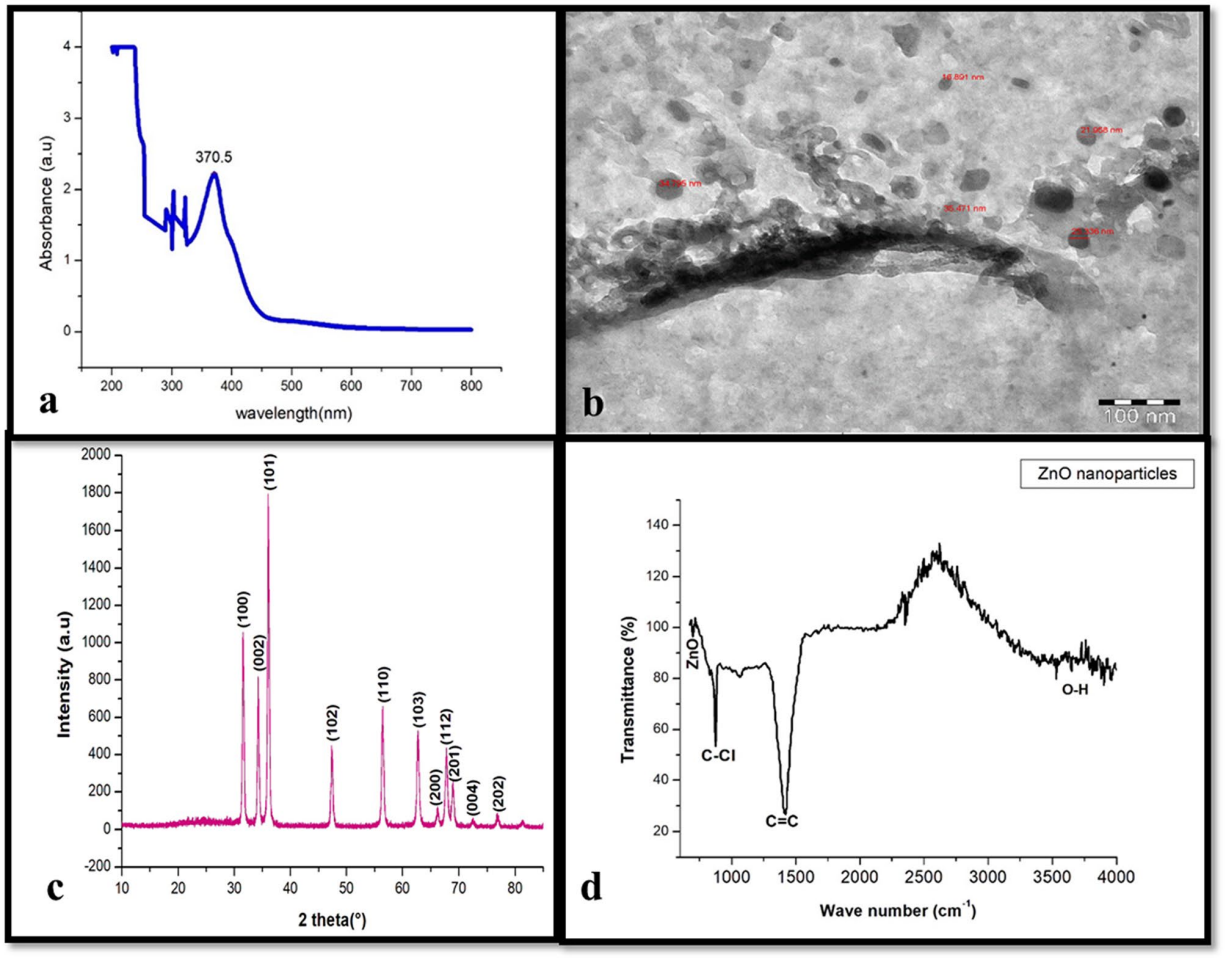
(**a**) UV-Visible absorption spectrum of the biosynthesized ZnO NPs (from 200 to 800 nm) band at 370.5 nm (**b**) TEM image of the biosynthesized ZnO NPs. Bar 100 nm (**c**) XRD Patterns of the biosynthesized ZnO NPs (from 10 to 85 2theta (°)) and (**d**) FTIR Spectrum of biosynthesized ZnO NPs (from 600 to 4000 cm^−1^).

The synthesis of biosynthesized ZnO NPs was carried out by taking 2:2 (v/v) of leaves extract of and zinc nitrate solution to obtain a mixture solution in a round-bottom flask, and incubated with constant stirring (100 rpm) at 40 °C for 24 h. The color of the reaction mixture was changed to yellow after 30 min of incubation time. The changing of color during the incubation time is the first sign of ZnO NPs formation. Then, the obtained biosynthesized ZnO Powder was submitted to various analytical techniques for characterization and to ascertain their shape, size and functionalization. The biosynthesized ZNO powder was dissolved in Milli-Q water to detected the UV-Visible spectra by using SHIMADZU SPECTROPHOTOMETER (UV-1800) in the range of 200–800 nm. The UV–Visible analysis showed that an absorption peak at 370.5 nm (Fig. 1a). No another major peak shifts had been observed during reactions and optimization. According to^38^ the range of UV spectrum for ZnO NPs was 368 nm. Moreover^39^, has been stated that, UV spectrum for ZnO NPs was observed at 375 nm. Additionally, many researchers reported that, UV Spectra of green zinc oxide nanoparticles is fluctuated between 360–380 nm. The shape and size of biosynthesized ZnO nanostructures was investigated using transmission electron microscope (TEM). TEM image of ZnO nanoparticles showed that, the particle size is ranged from 17 to 36 nm (Fig. 1b). XRD spectra of the biosynthesized ZnO powder was detected by X-ray diffractor, which resulted in different crystal planes such as (100), (002), (101), (102), (110), (103), (200), (112), (201), (004) and (202) were assigned to the 2θvalues of XRD 31.57°; 34.26°; 36.05°; 47.34°; 56.47°; 62.75°; 67.82°; 68.0°; 72.0°; 76.0° and 81.0° which showed hexagonal phase of zinc oxide and good crystallinity of the products (Fig. 1c). These presented planes are match well with the quartzite ZnO hexagonal structure having JCPDScard No. 36-1451 which was reported by^40^ (Fig. 1c). The FTIR analysis was performed to identified the chemical groups presented in the biosynthesized ZnO nanostructure. The band recorded at 675 cm^−1^ indicated that biosynthesized ZnO NPs well formed, the band at 876 cm^−1^ (C–Cl) with a stretch type of vibration, 1470 cm^−1^ (C=C) stretch mode of vibration, 1600 (N–H) bending vibration and 3200 to 3550 cm^−1^ attributed to the O–H mode of vibration and bending modes of the absorbed water. The obtained spectrum of FTIR analysis is represented in (Fig. 1d).

### The effect of biosynthesized ZnO NPs on biomass of *J. Procera*

**Figure 2:**
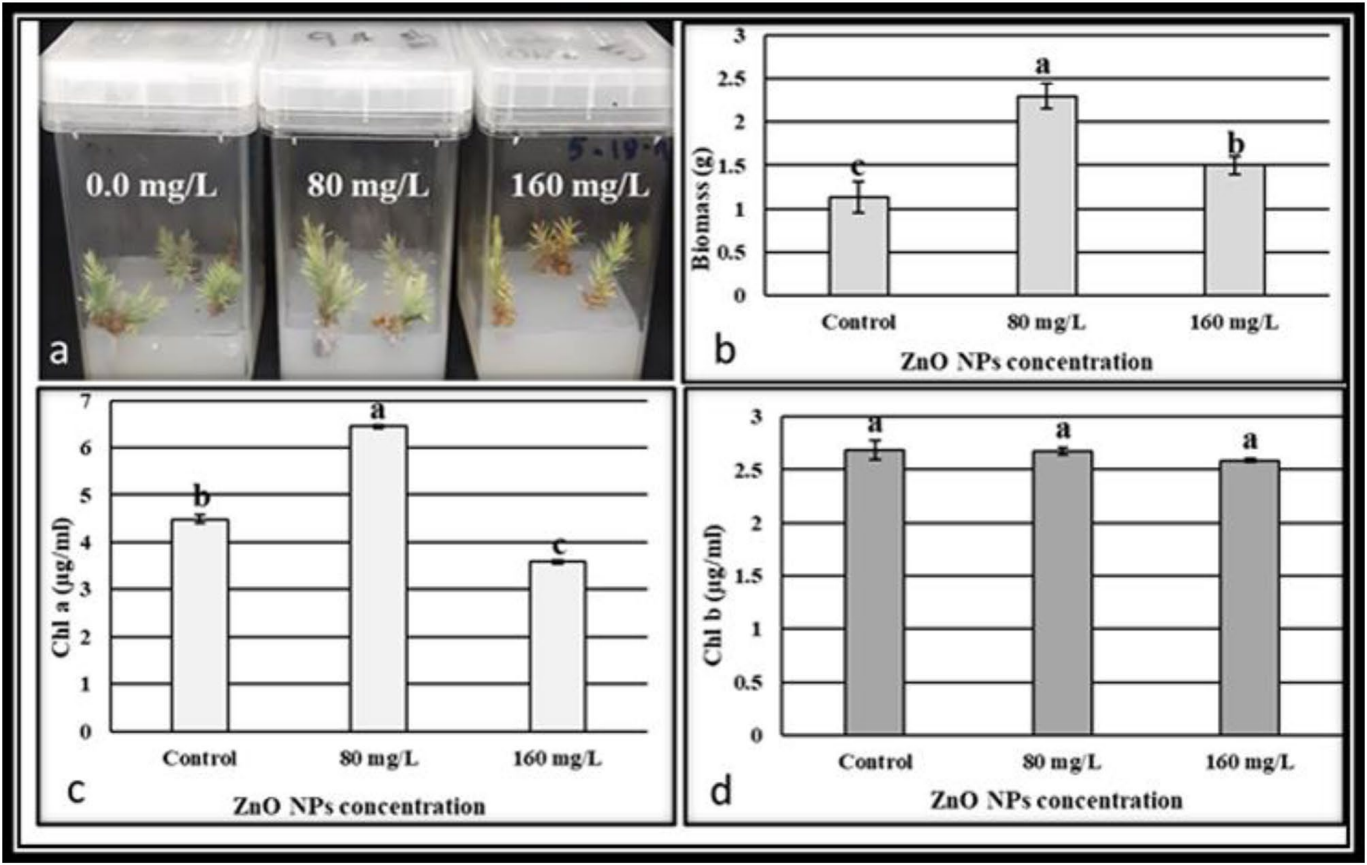
(**a**) Plants growth under different treatments of biosynthesized ZnO NPs (**b**) Biomass of plants under different treatments of biosynthesized ZnO NPs (g) (**c**) Chl a under different treatments of biosynthesized ZnO NPs (mg/ml) and (**d**) Chl b under different treatments of biosynthesized ZnO NPs (mg/ml). The data are presented the average of parameters ± SD. ^a,b,c^Means within the same column with different superscripts differ significantly (*P* < 0.05).

**Figure 3:**
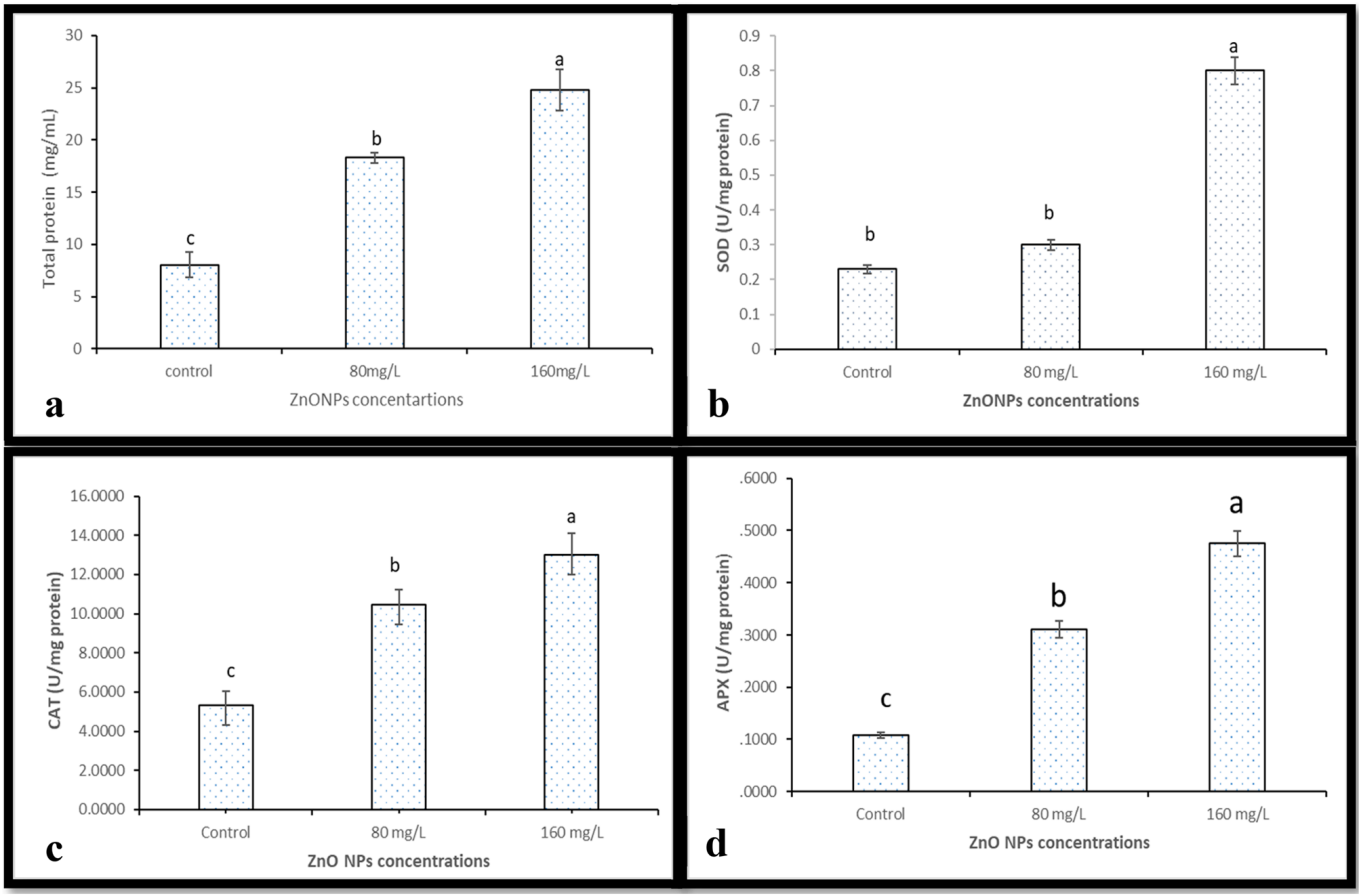
(**a**) Total protein contents under different concentration of ZnO NPs (**b**) SOD activity under different concentrations of ZnO NPs (**c**) CAT activity under different concentrations of ZnO NPs and (**d**) APX activity under different concentrations of ZnO NPs.

The effect of different concentrations (0.0 mg/L, 80 mg/L and 160 mg/L) of biosynthesized ZnO nanoparticles on biomass of *J. procera* growth in vitro was investigated after 70 day of growth. The obtained results indicated that, biosynthesized ZnO NPs concentrations (80 mg/L and 160 mg/L) were enhanced the growth of *J. Procera* significantly compared to non-treated plants (Fig. 2a,b). While, among ZnO NPs concentrations, the treatment of 80 mg/L biosynthesized ZnO NPs had the best biomass fresh weight (2.3 g) compared to 160 mg/L treatment (1.5 g) (Fig. 2a,b). Obviously, the addition of biosynthesized ZnO NPs to the plants media has improved regeneration of shoots and callus formation substantially. This result supported by physiological characterization which revealed that, ZnO NPs were increased the amount of chlorophyll a (Fig. 2c) and total protein content (Fig. 3 a) significantly compared to the control. In case of Chl b no significant result was recorded among different treatments (Fig. 2d).These findings were in accordance with^41^ who stated that, the presence of ZnO NPs was incorporated into plant hormone such auxin and improved plants growth. Chlorophyll an increased with significant result under ZnO NPs at 80 mg/L compared to control. Whereas at level (160 mg/L) was decreased significantly compared to control (Fig. 2c). May be due to toxic level of ZnO NPs, while no significant differences were recorded in case of chlorophyll b. The highest concentration (160 mg/L) of Biosynthesized ZnO NPs was enhanced growth of Plants at beginning, one month later the color of callus turned into brownish and shoots were changed to yellowish (Fig. 2a). Might be due high dose of zinc oxide nanoparticle, its seemed to be toxic. Although, zinc is an essential mineral at higher concentrations this metal is toxic mentioned by^42^. Moreover, ZnO NPs at high concentration inhibited the expression of genes involved in chlorophyll synthesis and photosystem structure^43^. Our findings in agreement with^44^ who has been reported that, plants treated with ZnO NPs nanoparticles showed significant growth compared to the control. Moreover, it was reported that, ZnO NPs play a major role in the increase in biomass, nutrients in wheat^45^. So far, ZnO NPs with low concentrations, it stimulated the callus growth and pointed out the nanoparticles role in regeneration, decontamination, organogenesis, callus induction and activated a protein that has a vital role in growth recounted by^14,15^. In turn, zinc oxide nanoparticles have potential to enhancement the growth and yield of crops^46,47^. A few studies have been focused on phytotoxicity and toxicological effect of ZnO NPs on plants. In general, studies with NPs indicated a certain degree of phytotoxicity, especially at high concentrations^48^. Exposure plants to NPs have induced reactive nitrogen species^49^. Moreover, the highly concentration of ZnO NPs in the rhizosphere solution and root surface could potentially impact the ryegrass growth stated by^50^. Recent studies have shown that plant growth, development and physiology are significantly affected by nanoparticles. Finally, the lowest values of plants biomass were recorded in non-treated plant (Fig. 2a,b).

### The impact of ZnO NPs on Protein contents and enzymes activity of callus of *J. procera *in vitro

#### Total protein contents

The total protein of callus of *J. procera* was determined using Nano drop. The results showed that, there were significant differences in total protein contents among callus treated with different concentrations of ZnO NPs. While, among the ZnO NPs treatments, the highest concentration of biosynthesized ZnO NPs (160 mg/L) has given the highest level of total protein (Fig. 3a and Table 1). In accordance, ZnO nanoparticles have increased protein content in tomato even under salt stress, stated by^41^. Moreover^51^ who has been reported that, ZnO nanoparticles have a positive effect on protein content of callus of *Nicotiana tabacum*. ZnO NPs cause a great effect on expression of some genes encoding certain proteins, it could be caused turn on or turn off the expression of some genes reported by^52^. Also, it is suggested that ZnO nanoparticles might be provide the plants with bio-available Zn ion at cellular level.

**Table 1:** Effect of ZnO NPs on total protein, enzymes activity, proline, flavonoids and total phenolic contents of *J. procera.*

ZnO NPs treatments	Total protein (mg/ml) ± SD	SOD (U/mg protein) ± SD	CAT (U/mg protein) ± SD	APX (U/mg protein) ± SD	Proline (mg/g. F. wt) ± SD	Total flavonoids (mg/g dry.wt) ± SD	Total phenolic contents (mg/ml) ± SD
0.0 mg	8.03 ± 1.20^c^	0.23 ± 0.04^b^	5.32 ± 0.72^c^	0.10 ± 0.01^c^	126 ± 30^c^	23.31 ± 1.78^b^	104.8 ± 0.11^b^
80 mg	18.30 ± 0.51^b^	0.3 ± 0.09^b^	10.47 ± 0.74^b^	0.31 ± 0.05^b^	386 ± 30^b^	28.43 ± 0.80^a^	74.5 ± 0.11^c^
160 mg	24.79 ± 1.94^a^	0.4 ± 0.06^a^	13.02 ± 1.1^a^	0.47 ± 0 .04^a^	580 ± 90^a^	29.37 ± .0.40^a^	133.9 ± 0.2^a^

The data are presented the average of total protein, enzymes activity, proline, total flavonoids and total phenolic contents ± standard deviation (SD).

^a,b,c^Means within the same column with different superscripts differ significantly (*P* < *0.05*).

#### Superoxide dismutase

The influence of ZnO nanoparticles on SOD activity in callus of *J. procera* under different treatments was investigated as an important scavenger for reactive oxygen species (ROS). SOD activity was induced by highest levels (160 mg) of ZnO NPs in the medium compared to untreated control significantly (Fig. 3b and Table 1). It has been stated that, zinc oxide nanoparticles was increased the activity of SOD in *Punica granatum* callus^53^ which is in agreement with our findings in this study. This may be due to The regulations in SOD in response to stress which might be caused by nanoparticles, SOD is well-known as powerful ROS Scavenger.

#### Catalase

The effect of ZnO NPs on CAT activity in callus of *J. procera* exposed to different levels of ZnO NPs was evaluated. The result indicates that; the activity of CAT was stimulated with the increased levels of ZnO NPs significantly compared non-treated-control. On the other hand, callus was significantly affected by ZnO nanoparticles. Additionally, the result showed that CAT activity in the case of NPs represented strong correlations with ZnO NPs (Fig. 3c and Table 1). In this context^53^, who has been reported that, zinc oxide nanoparticles were increased the CAT activity in callus of pomegranate.

#### Ascorbate peroxidase

APX activity was assessed as it catalyzes the hydrogen peroxide dependent oxidation of ascorbate in callus under different treatments of ZnO nanoparticles. The results reveled that, the increasing ZnO NPs levels were increased APX activity significantly compared to non-treated callus. Moreover, under ZnO NPs treatments, APX activity showed a strong positive association ZnO NPs concentrations (Fig. 3d and Table 1). APX activity was induced by zinc oxide nanoparticle and has strong association with ZnO NPs concentrations reported by^53^.

#### Proline

**Figure 4:**
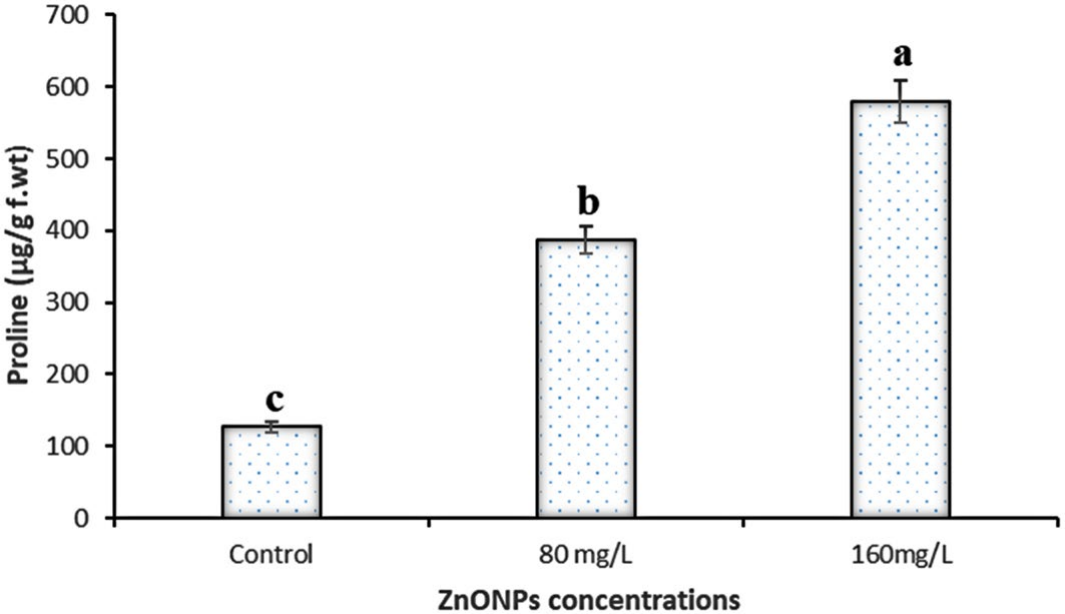
The effect of different concentrations of ZnO nanoparticles on proline in callus of *J. procera.*

Proline (Pro) accumulation is physiological response in many plants to a wide range of abiotic and biotic stresses and can be a reactive oxygen species scavenger^54^. The examined of pro in this study showed that, the ZnO NPs have increased the level of pro in callus of *J. procera* significantly compared to non-treated plants. The increasing of the pro has strongly correlation with the increasing of nanoparticles (Fig. 4 and Table 1). ZnO nanoparticles might be induced plant to form proline. It has been reported that, the addition of ZnO NPs to plants have increased proline contents significantly^55^.

#### Total flavonoids

**Figure 5:**
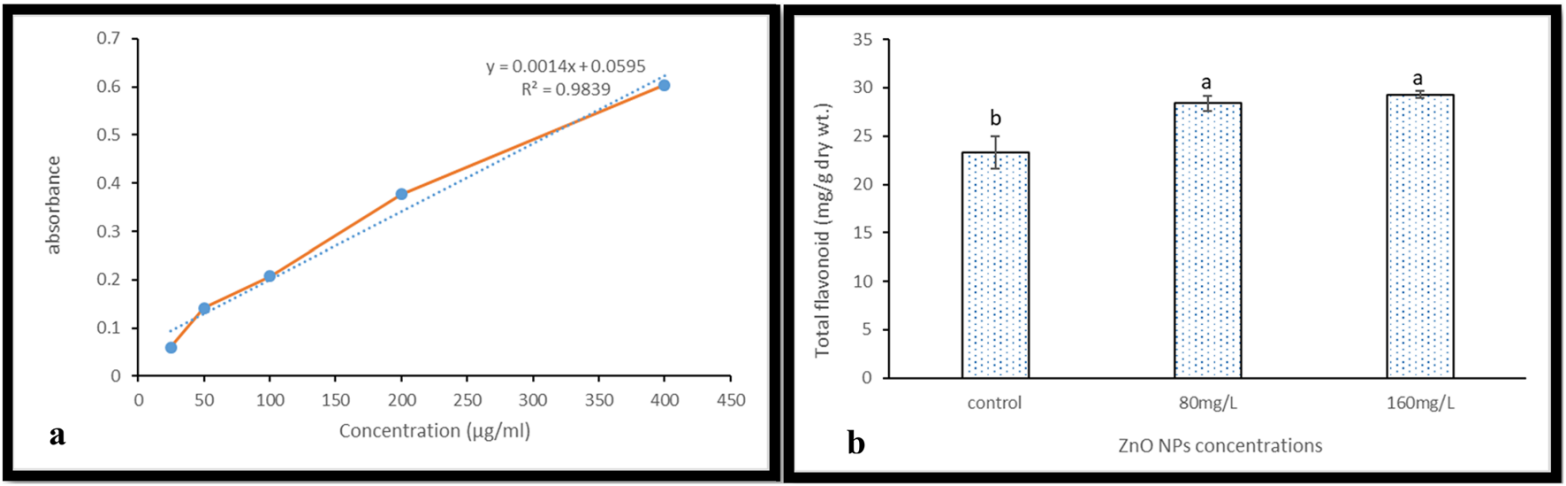
(**a**) Quercetin standard curve (**b**) the impact of Zno nanoparticles on total flavonoids (mg/g dry.wt).

Flavonoids are secondary metabolites with antioxidant activity, the potency of which depends on the number and position of free OH groups^56^. Flavonoids have many biological activities such as the treatment of asthma, bronchitis and cardiovascular disease, the improvement of peripheral blood flow and reduction of cerebral insufficiency^57^. Here, in this study, the estimation of total flavonoid content of callus extract of *J. procera* was carried out by using Uv-spectrophotometer and quercetin as calibration curve (y = 0.0014x + 0.0595, R2 = 9839) (Fig. 5a and Table 1). The results showed that, biosynthesized zinc oxide nanoparticles have increased the total flavonoids in treated callus of *J. procera* significantly compared to non-treated callus (Fig. 5b). ZnO nanoparticles could be cause stress to the plants which lead to accumulation of flavonoids to act as scavengers. Higher content of flavonoids and phenol was observed in ZnO NPs as compare to crude extract was reported by^58^.

#### Total phenolic contents

**Figure 6:**
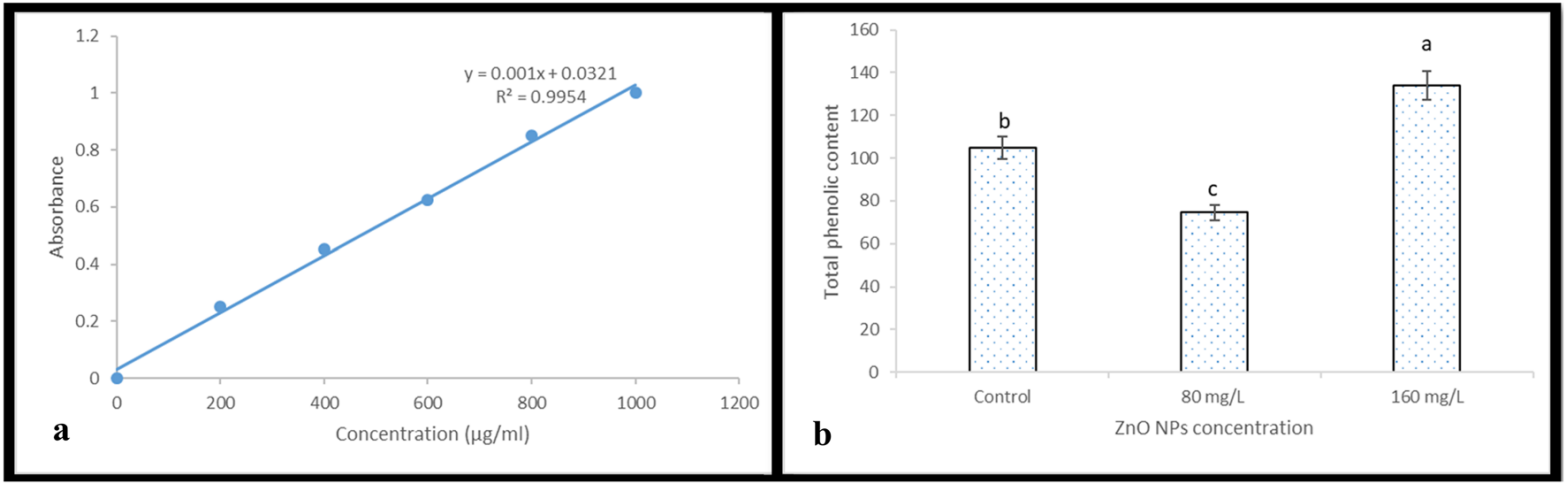
(**a**) Gallic acid standard curve. The standard curve is used to estimate phenolics (gallic acid equivalents) in a 200-mlsample. (**b**) Total phenolic content under different treatments of biosynthesized ZnO NPs.

Phenols are excellent oxygen radical scavengers because the electron reduction potential of the phenolic radical is lower than the electron reduction potential of oxygen radicals^59,60^, and also because phenoxyl radicals are generally less reactive than oxygen radicals^61^. Therefore, phenolic compounds can scavenge reactive oxygen intermediates without promoting further oxidative reactions^59^. The total phenolic content in callus of *J. procera* was determined using Uv-spectrophotometer and Gallic acid was used as a reference standard calibration curve (Fig. 6a). This result indicates that, ZnO NPs nanoparticles at highest level have induced the formation of phenolic content significantly compared control (Fig. 6b and Table 1). During the stress caused by heavy metals, phenolic compounds act as metal chelators and accumulated. Hence, increases in antioxidant activity of plants exposed to NPs, is mainly due to the increase in phenolic compounds, which are ROS scavengers^62^.

#### The effect of biosynthesized ZnO NPs on bioactive compounds production

**Figure 7:**
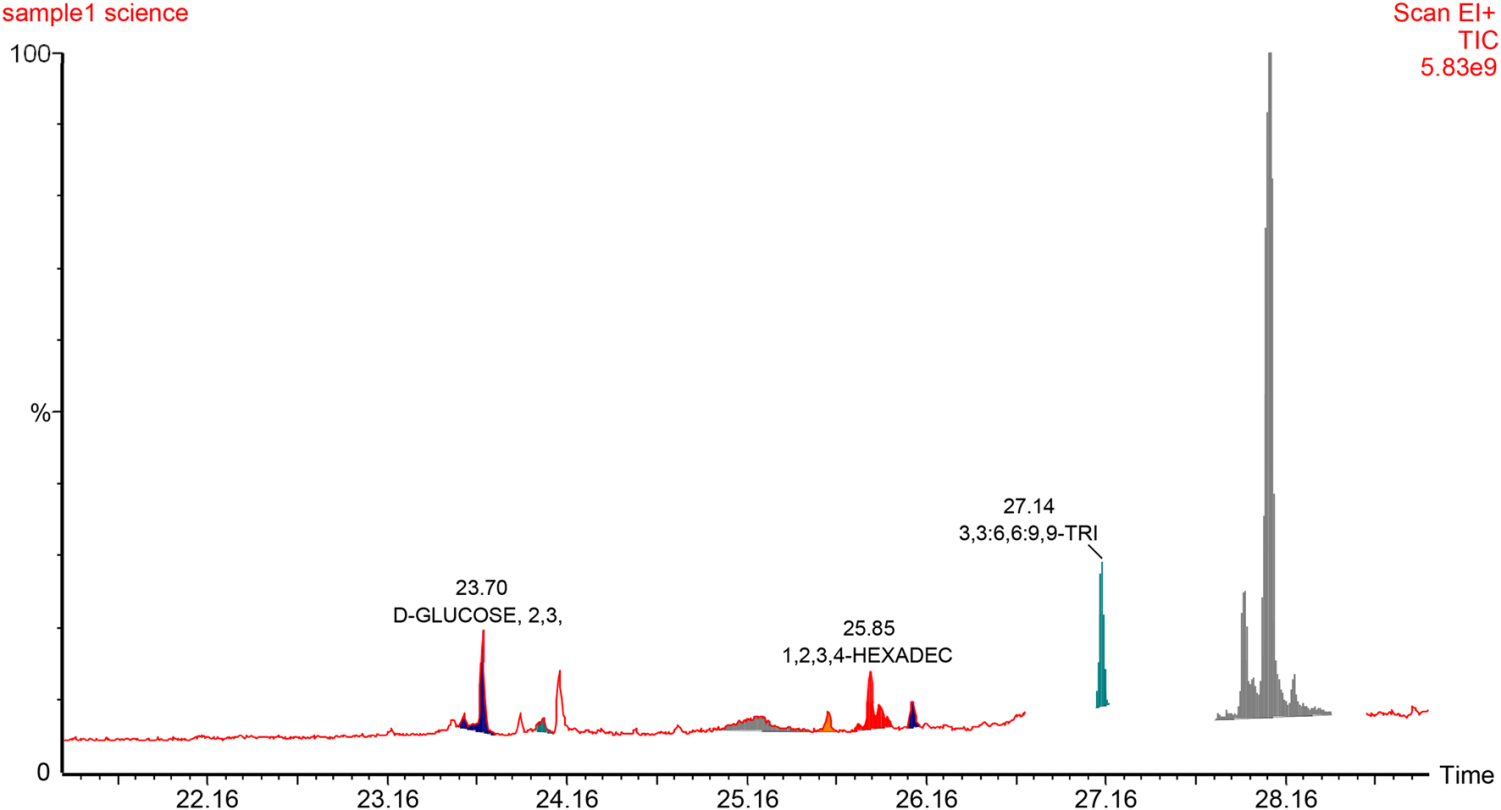
GC–MS analysis chromatograms of non-treated callus of *J. procera.*

**Figure 8:**
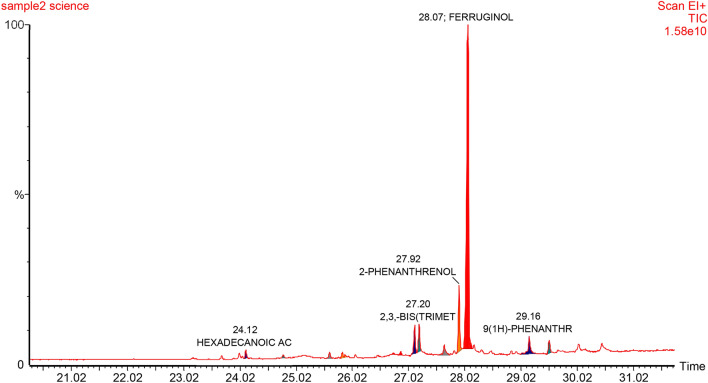
GC–MS analysis chromatograms of callus of *J. procera* under treatment of ZnO NPs (80 mg/L).

**Figure 9:**
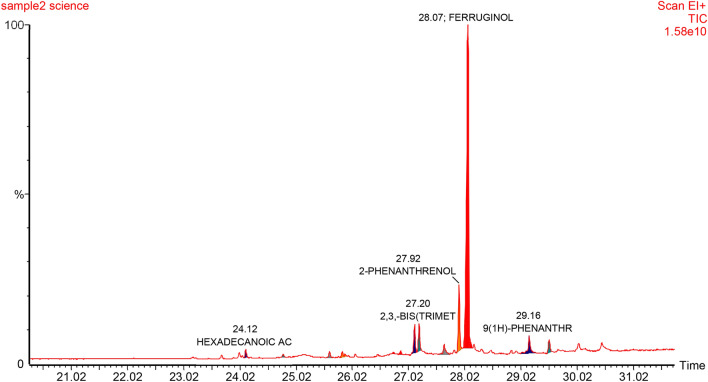
GC–MS analysis chromatograms of callus of *J. procera* under treatment of ZnO NPs (160 mg/L).

In nature, plants produce bioactive compounds as a protection mechanism against abiotic and biotic stress, and attraction or signaling. Phytochemicals have been used by human as condiments, flavorings and for treating health disorders and preventing diseases including epidemics. The availability of some phytochemicals constituents from its current natural sources are limited. Hence, alters and inducers agents are needed to increase the productivity of bioactive compounds or even to generate new ones. As in nature, plants produce these products as repose to stress. Therefore, plants have been exposed to different concentrations of biosynthesized zinc oxide nanoparticles. The effect of ZnO NPs on bioactive compounds in callus of *J. procera* was investigated. The differences have been observed among the treatments. The main phytochemical compound in methanol extract of callus of *J. procera* under different ZnO nanoparticles concentrations is ferruginol (Table 2 and Figs. 7, 8 and 9). To date, there are no reports comparing the effect of biosynthesized ZnO NPs on the production and accumulation of bioactive compounds in callus of *J. procera.* The result indicates that, nanoparticles have impacted on secondary metabolites production and was significantly affected. Nanoparticles could be promoting formation of some phytochemicals as well as inhibit others. Moreover, plants develop resistance to metal stress by altering phytochemicals accumulation and certain antioxidant enzymes to counteract oxidative damages of cellular components and biomolecules caused by highly reactive free radicals^63^. The effect of nanoparticles on plant secondary metabolism still obscure^16^. Therefore, it is a priority to understand the impact of nanoparticles on secondary metabolites which might be helped in production process and it can be used as promotors.

**Table 2:** The effect of different concentrations of biosynthesized zinc oxide nanoparticles on bioactive compounds of callus from *J. procera.*

Callus (0.0 mg/L ZnO NPs)—compounds	RT (min)	Area %	Area
D-glucose	23.80	4.270	351,486
D-mannitol	24.21	28.270	2,324,943
Hexadecanoic acid	25.20	31.670	2,605,308
1,2,3,4-hexadecanetetrol	25.90	3.920	322,658
Methyl ester of octadecanoic acid	26.10	9.100	748,429
Ferruginol	28.06	20.990	1,726,410
**Callus (80 mg/L ZnO NPs)—compounds**			
Methyl ester of hexadecanoic acid	24.16	53.980	458,282
4B,5,6,7,8A,9,10-heptahydro-phenanthrane	25.62	0.200	1724
Methylester of 3-cyclohexyl-propionic acid	25.84	24.980	212,040
Methylester of 3-cyclohexyl-propionic acid	25.84	24.980	212,040
Totarol	27.99	6.000	50,937
Ferruginol	28.00	10.190	86,498
**Callus (160 mg/L ZnO NPs)—compounds**			
Methyl ester of hexadecanoic acid	24.09	37.010	553,334
4b,5,6,7,8a,9,10-heptahydro-phenanthrane	25.66	7.740	115,641
5-methyl-5h-benzo[b]furo[2,3-d]oxepin-4-one	27.23	24.320	363,528
4b,5,6,7,8,8a,9,10-octahydro-4b,8,8-trimethyl-1-(1-methylethyl)2-phenanthrenol	28.09	4.210	63,006
Ferruginol	28.20	8.290	123,918
1,2,3,4,4a,9,10,10a-octahydro-7-methoxy-1,1,4 a-trimethyl-8-(1-methylethyl)phenanthrene	28.99	0.380	5640

## Conclusion

In summary, it could be concluded that, the addition of biosynthesized ZnO nanoparticles to the media of plants in vitro had played vital role in biomass production. ZnO nanoparticles had greater and more-responsive effect on the *J. procera* shoots and calli growth and physiological indices compared to non-treated plants. Which resulted in higher growth of callus, shoots, chlorophyll, total protein content, total phenols and flavonoids contents and enzymes activity. Obviously, this study showed that, ZnO nanoparticles simultaneously induced growth promoting or factor can cause oxidative stress effects in the plant cells depends on concentrations of ZnO nanoparticles. However, our knowledge of the influence of nanoparticles on living systems is mostly inconsistent to date. The increasing number of studies on nanoparticles has been focused on nanoparticles bio-availability with respect to their concentration. Moreover, the influence of nano material is widely investigated under in vivo systems, as elicitors accompanied by biotic or abiotic conditions. But the effect of nanoparticles on bioactive compounds production in vitro is requested to be developed and validated. Therefore, we suggested that, the effect of nanoparticle on bioactive compounds should be to elucidated. The result of current study could be concluded that, the nature of nanoparticle-derived Zn ions might have had a more significant effect when other factors are taken into consideration, such as doses, particle size, particle and concentration and growth media. Further, the study which is designed and presented in this paper can be extended to involve quantification of bioactive compounds and biosynthesis pathways of the important phytochemical compounds can be tested under ZnO nanoparticles treatment, which might be lead to better understanding of the effect of nanoparticles on antioxidant systems of plants.

## Data Availability

The data used or analyzed during the present study are available from the corresponding author/KSU.
